# [^68^Ga]-Pentixafor PET/CT for CXCR4-Mediated Imaging of Vestibular Schwannomas

**DOI:** 10.3389/fonc.2019.00503

**Published:** 2019-06-12

**Authors:** Maria Breun, Camelia M. Monoranu, Almuth F. Kessler, Cordula Matthies, Mario Löhr, Carsten Hagemann, Andreas Schirbel, Steven P. Rowe, Martin G. Pomper, Andreas K. Buck, Hans-Jürgen Wester, Ralf-Ingo Ernestus, Constantin Lapa

**Affiliations:** ^1^Department of Neurosurgery, University Hospital Würzburg, Würzburg, Germany; ^2^Department of Neuropathology, University of Würzburg, Institute of Pathology, Würzburg, Germany; ^3^Comprehensive Cancer Center (CCC) Mainfranken, Würzburg, Germany; ^4^Department of Nuclear Medicine, University Hospital Würzburg, Würzburg, Germany; ^5^Division of Nuclear Medicine and Molecular Imaging, The Russell H. Morgan Department of Radiology and Radiological Science, Johns Hopkins University School of Medicine, Baltimore, MD, United States; ^6^Pharmaceutical Radiochemistry, Technische Universität München, Munich, Germany

**Keywords:** vestibular schwannoma, CXCR4, PET/CT, molecular imaging, Pentixafor

## Abstract

We have recently demonstrated CXCR4 overexpression in vestibular schwannomas (VS). This study investigated the feasibility of CXCR4-directed positron emission tomography/computed tomography (PET/CT) imaging of VS using the radiolabeled chemokine ligand [^68^Ga]Pentixafor.

**Methods:** 4 patients with 6 primarily diagnosed or pre-treated/observed VS were enrolled. All subjects underwent [^68^Ga]Pentixafor PET/CT prior to surgical resection. Images were analyzed visually and semi-quantitatively for CXCR4 expression including calculation of tumor-to-background ratios (TBR). Immunohistochemistry served as standard of reference in three patients.

**Results:** [^68^Ga]Pentixafor PET/CT was visually positive in all cases. SUV_mean_ and SUV_max_ were 3.0 ± 0.3 and 3.8 ± 0.4 and TBR_mean_ and TBR_max_ were 4.0 ± 1.4 and 5.0 ± 1.7, respectively. Histological analysis confirmed CXCR4 expression in tumors.

**Conclusion:** Non-invasive imaging of CXCR4 expression using [^68^Ga]Pentixafor PET/CT of VS is feasible and could prove useful for *in vivo* assessment of CXCR4 expression.

## Introduction

Vestibular schwannomas (VS) are benign nerve sheath tumors that arise from Schwann cells of the vestibulocochlear nerve (1, 2). VS regularly cause hypoacusis, dizziness, and tinnitus. These tumors usually arise sporadically, however in ~5% of the cases they are associated with a rare (1:33,000) genetic disorder, neurofibromatosis type 2 (NF2). In NF2, various types of tumors including schwannomas, meningiomas, and ependymomas develop due to loss of the NF2 gene, which encodes for Merlin, a tumor suppressor protein (3, 4).

VS are the hallmark tumors of this disease. In NF2, they usually appear bilaterally, and compared to sporadic schwannomas, they grow faster and are much more adherent to the cranial nerves and the brainstem (5–7). Accordingly, NF2-associated vestibular schwannomas are the more aggressive tumor entity. Surgery is the standard treatment in sporadic schwannoma, but not a long-lasting solution for NF2-related tumors since the disease is often associated with persistent cranial nerve deficits and high recurrence rates. Thus, efficacious systemic or non-invasive therapies would be of value for these patients.

Chemokines are important regulators of the tumor environment, which, in addition to Merlin loss in Schwann cells, is essential for tumor development in VS. C-X-C motif chemokine receptor 4 (CXCR4), a 40-kDa G protein-coupled receptor of the chemokine receptor subfamily, was initially found to regulate leukocyte trafficking (8–11). It plays an important role in the process of homing and recruitment of progenitor and immune cells, and it is integral to the development of the nervous, hematopoietic, and cardiovascular systems during embryogenesis (9, 10, 12). However, it is also involved in diverse pathological processes, including infection, autoimmune disease, and cancer (9, 13). CXCR4 overexpression has been described in more than 30 different tumor entities including breast, prostate, lung, and colon cancer, as well as in neuroblastoma and peripheral nerve sheath tumors (10, 14). Significant overexpression of CXCR4 in both sporadic as well as neurofibromatosis-associated VS was recently demonstrated (15, 16). Therefore, CXCR4 could serve as a new target for systemic therapy with specific inhibitors (e.g., AMD3100) (17, 18). CXCR4 inhibitors have already been approved for leukemia therapy and are under investigation in trials for several solid tumors (19–21).

The radiolabeled CXCR4-targeted ligand [^68^Ga]Pentixafor was recently developed for PET imaging (22). CXCR4 expression has been demonstrated in multiple types of cancer, including adrenocortical carcinoma (23), SCLC, glioblastoma, and hematologic malignancies (24–27). This manuscript is the first report of non-invasive detection of CXCR4 expression in patients with sporadic and NF2-associated VS.

## Materials and Methods

### Patients

From June to December 2017, a total of four patients with either newly diagnosed (*n* = 1) or pre-treated and observed VS (*n* = 3) underwent imaging with [^68^Ga]-Pentixafor-PET/CT. Routine diagnosis before surgery included MRI and was available in all patients. Six tumors were VS and one was a facial nerve schwannoma. Patient characteristics regarding tumor extension and clinical impairment are given in more detail in Table 1. [^68^Ga]-Pentixafor was administered on a compassionate use base in compliance with §37 of the Declaration of Helsinki and the German Medicinal Products Act, AMG §13 2b, and in accordance with the responsible regulatory body (Regierung von Oberfranken). All patients gave written, informed consent prior to imaging. Due to the retrospective nature of this study, the local institutional review board (University Hospital Würzburg, Würzburg, Germany) waived the requirement for additional approval.

**Table 1:** Tumor characteristics and imaging results.

**No**	**Tumor location**	**Tumor extension**	**Antoni type**	**Ki 67 (%)**	**CXCR4 IRS**	**SUV_**max**_**	**TBR_**max**_**	**TBlR_**max**_**
1	VS left	T3A 16 × 8 mm	A/B	1	9	3.37	3.59	1.32
2	VS right	T4B 30 × 35 mm	A	1–2	2–6	4.13	4.17	1.59
3	VS left	T4A 23 × 23 mm				3.78	6.87	1.71
	VS right	T3B 16x12 mm		None		4.05	7.36	1.83
4	VS left	T4 26 × 32 mm	A/B	10–15	6	3.26	3.62	1.62
	VS right	T3A				3.99	4.43	1.99

*IRS, immunoreactive score; SUV, standardized uptake value; TBlR, tumor to blood pool ratio; TBR, tumor to background ratio; VS, vestibular schwannoma*.

### Imaging and Image Analysis

All PET scans were performed on a dedicated (PET/CT) scanner (Siemens Biograph mCT 64; Siemens Medical Solutions, Erlangen, Germany). [^68^Ga]Pentixafor PET was performed on the day prior to surgery, 60 min after i.v. injection of 88 to 163 MBq (mean: 135 ± 28 MBq) Low-dose CT scans of the brain for attenuation correction were acquired (35 mAs, 120 keV, a 512 × 512 matrix, 5 mm slice thickness, increment of 30 mm/s, rotation time of 0.5 s, and pitch of 0.8). All PET images were iteratively reconstructed (3 iterations, 24 subsets with resolution recovery; Gaussian filtering: 2 mm; matrix: 400 × 400) using corrections for attenuation, dead-time, random events, and scatter. Acquisition and data reconstruction were performed using dedicated manufacturer software (syngo MI.PET/CT; Siemens Healthineers, Erlangen, Germany).

Images were first inspected visually by a reader with expertise in the interpretation of [^68^Ga]Pentixafor PET (C.L.). Then the axial PET image slice displaying the maximum tumor uptake was selected. Tumor regions of interest (ROIs) were defined in 2 ways. First, a standardized 10-mm circular region was placed over the area with the maximum activity. This first ROI was used to derive maximum (SUV_max_) and mean standardized uptake values (SUV_mean_). A normal reference brain region was defined by drawing a ROI (diameter of 25 mm) involving the contralateral cerebral hemisphere at the level of the centrum semiovale to derive tumor-to-background ratios. Additionally, another ROI (3D isocontour) was placed in the superior sagittal sinus (at the tumor level) to derive an estimate of blood pool activity (for respective tumor-to-blood pool ratios). The radiotracer concentration in the ROIs was normalized to the injected dose per kilogram of patient's body weight to derive the SUVs.

### Immunohistochemistry

All tumors were histologically assessed and graded on formalin fixed and paraffin embedded tissue sections by an experienced neuropathologist (CMM) according to the 2016 criteria of the World Health Organization (2). Schwann cell origin of the tumor cells was confirmed by the positive reaction with S100 antiserum (1:200, Dako, Hamburg, Germany). To determine the proliferative activity of tumor cells, the Ki-67 labeling index was calculated after immunostaining (monoclonal, clone Ki-67, 1:50, Dako, Hamburg, Germany) by determining the number of positive nuclei among 100 tumor cells per high power field (HPF) (x400) in a total of 10 HPF per sample.

The VS sections (3 μm) were cut from formalin-fixed paraffin-embedded tissue blocks and stained with anti-CXCR4 antibody (Zytomed 503-18440, Berlin, Germany) using a 1:50 dilution in dilution buffer (DCS, Jena, Germany). CXCR4 protein expression was visualized using a poly-link secondary antibody and a peroxidase kit (Dako; DCS Innovative Diagnostic Systems, Jena, Germany). Positive signals resulted in brown staining, and counterstaining was performed with hematoxylin. All immunohistochemically stained slides were analyzed using light microscopy (Leica, Wetzler, Germany). Negative control experiments were performed by staining low-grade astrocytomas, and positive control experiments were performed by staining placenta sections with primary and secondary antibodies.

The analysis of the stained sections was done semi-quantitatively by light microscopy according to the immunoreactive score (IRS) described by Remmele and Stegner (28). The percentage of CXCR4 positive cells was scored as follows: 0 (no positive cells), 1 (<10% positive cells), 2 (10–50% positive cells), 3 (>50–80% positive cells), 4 (>80% positive cells). Additionally, the intensity of staining was graded: 0 (no color reaction), 1 (mild reaction), 2 (moderate reaction), 3 (intense reaction). Multiplication of both scores for a given sample yielded the IRS classification: 0–1 (negative), 2–3 (mild), 4–8 (moderate), 9–12 (strongly) positive.

### Statistical Analysis

Statistical analyses were performed using Graph Pad Prism 6 software (GraphPad Software, La Jolla, CA, USA). For descriptive statistics, quantitative values were expressed as mean ± standard deviation or median and range as appropriate. Comparisons of related metric measurements were performed using the two-tailed *t*-test. A *p* <0.05 was considered to indicate statistical significance.

## Results

### Clinical Data

Two patients (patients #3 and #4) had neurofibromatosis type 2 with bilateral VS which had been previously treated with (radio-)surgery. In the remainder, VS were unilateral with a subject with newly diagnosed (patients #2) and a single patient with known but untreated tumors (patient #1).

Two of six tumors had been previously treated with radiosurgery and one with surgery. The remaining three tumors had no prior treatment. Three tumors were progressive as described by consecutive MR imaging with a growth rate higher than the average of 2 mm per year (29, 30), one tumor was newly diagnosed and directly treated after diagnosis. Two tumors were stable regarding their extension after radiosurgery, but caused progressive hearing impairment necessitating further treatment. Patients‘ characteristics are displayed in Table 1 and Supplemental Table 1.

### Imaging Results and Analysis

**Figure 1:**
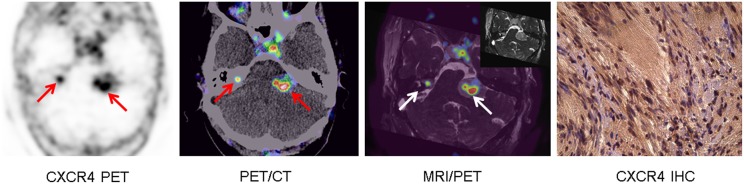
Example of increased CXCR4 expression in bilateral vestibular schwannoma in NFII (patient #4). Display of transaxial CXCR4-directed positron emission tomography (PET), fused PET/computed tomography (CT), three-dimensional constructive interference in steady state (T2 CISS) magnetic resonance imaging (MRI; insert) and fused MRI/PET slices (arrows), which show increased chemokine receptor expression of bilateral vestibular schwannomas (arrows). Immunohistochemistry (IHC) of the left-sided tumor confirmed chemokine receptor expression [moderate (Score: 2) CXCR4 expression in 57% of tumor cells (Score: 3) resulting in an immunoreactive score of 6].

All VS demonstrated enhanced [^68^Ga]Pentixafor uptake (7/7, 100.0%). All lesions were visually clearly delineated from normal brain tissue and adjacent structures. Of note, the NF2 patients displayed radiotracer accumulation in all VS (Figure 1), irrespective of prior treatment.

SUV_mean_ and SUV_max_ were 3.0 ± 0.3 and 3.8 ± 0.4, respectively. With background SUV_mean_ of 0.8 ± 0.2, TBR_mean_ was 4.0 ± 1.4 and TBR_max_ was 5.0 ± 1.7. Blood pool activity ranged between 2.0 and 2.6 (median, 2.4; mean, 2.3 ± 0.3) and was significantly lower than VS SUV (*p* < 0.05). Individual imaging results can be found in Table 1 and Supplemental Table 2.

### Immunohistochemistry Analysis

Immunohistological evaluation of CXCR4 expression for comparison with imaging results was available for three patients. In all samples, CXCR4 was detectable at the cell membrane and in the cytoplasm.

Patient #1 (maximum tumor extension: 16 × 8 mm) demonstrated intense (Score: 3) membranous CXCR4 expression in 52% of cells (Score: 3) which results in an IRS of 9. Patient #2 (maximum tumor extension: 30 × 35 mm) showed areas with mild CXCR4 expression in 37.5% of schwannoma cells and areas with moderate CXCR4 expression in 55.5% of tumor cells. Accordingly, IRS of 2 for the low-expression and of 6 for the moderate-expression areas were calculated. Patient #4 (maximum tumor extension: 26 × 32 mm) presented moderate (Score: 2) cell surface CXCR4 expression in 57% of tumor cells (Score: 3) resulting in an IRS of 6 (Figure 1).

In this limited cohort, there was no correlation of immunohistochemistry to the Ki67 proliferation index or [^68^Ga]Pentixafor PET uptake.

## Discussion

This is the first report of *in vivo* imaging of CXCR4 expression in humans with VS. A recent report from our group evaluating samples of these tumors had demonstrated higher chemokine receptor expression in VS as compared to healthy vestibular nerves, with higher CXCR4 expression levels trending to correlate with greater functional impairment (16). In concordance with *in vitro* data, receptor expression on the cell surface was visualized by [^68^Ga]-Pentixafor PET/CT in our cohort in all cases, even in VS as small as 8 mm.

Considering the high tumor recurrence rate and frequently debilitating functional outcomes of patients after VS resection, especially in NF2 cases, a new therapeutic approach would be of tremendous value. Given that all tumor lesions in our cohort demonstrated CXCR4-positivity, CXCR4 could be a promising target for chemokine receptor-directed therapies. We have found that treatment with CXCR4 antagonists reduces schwannoma growth in cell culture experiments (unpublished data). Given the commercial availability of specific chemokine receptor inhibitors such as AMD3100, systemic blockage of CXCR4 might be a promising approach to (NF2-associated) VS treatment.

An important pre-requisite for receptor-targeted therapy is robust expression of the target and the possibility of *in vivo* imaging to select patients who are most appropriate for the treatment. PET imaging with the radiolabeled CXCR4 ligand [^68^Ga]Pentixafor has already proven its value for the non-invasive visualization of receptor expression in a number of various tumor entities (25, 31). In this pilot cohort, [^68^Ga]Pentixafor PET/CT was able to detect all schwannomas with sufficient tumor-to-background and tumor-to-blood pool ratios and matched with membranous CXCR4 expression as assessed by immunohistochemistry. Thus, CXCR4-directed PET/CT might serve as a non-invasive, *in vivo* read-out for identification of potential candidates for targeted therapy. Future studies might also investigate the relationship between CXCR4 and somatostatin receptors, which have also been demonstrated to be expressed in peripheral nerve sheath tumors and might therefore represent another suitable option (32).

This pilot study has several limitations. First, only a limited number of patients could be included in the study, thus precluding any robust conclusions from this cohort. Second, histological data for comparison with imaging findings were available in only three cases and no correlation of [^68^Ga]Pentixafor uptake with histological receptor expression could reasonably be calculated because of the limited number of cases. Autoradiography was not performed.

In the future, larger studies should be carried out to fully explore the binding of [^68^Ga]Pentixafor to membranous CXCR4 and to assess whether CXCR4-directed therapy is a viable option for patients with VS.

## Conclusion

Our pilot data demonstrate the feasibility of non-invasive imaging of CXCR4 expression in VS. [^68^Ga]Pentixafor PET/CT could prove to be a useful tool for *in vivo* assessment of CXCR4 expression, especially in NF2-mutated patients. Further research to elucidate the biologic implications and potential role of [^68^Ga]Pentixafor PET in selecting patients for CXCR4-directed therapy is warranted.

## Data Availability

All datasets generated for this study are included in the manuscript and/or the Supplementary Files.

## Ethics Statement

[68Ga]-Pentixafor was administered on a compassionate use base in compliance with §37 of the Declaration of Helsinki and the German Medicinal Products Act, AMG §13 2b, and in accordance with the responsible regulatory body (Regierung von Oberfranken). All patients gave written, informed consent prior to imaging. Due to the retrospective nature of this study, the local institutional review board waived the requirement for additional approval.

## Author Contributions

MB and CL participated in the design of the study. MB and CL performed the experiments and performed the data analysis and interpretation. ML, CM, and CMM provided the samples. MB and CL coordinated the work and drafted the manuscript, with the help of and critical revision by CM, R-IE, SR, MP, ML, AB, and CH. AFK and AS helped in performing the experiments. H-JW gave technical and pharmaceutical input. All authors read and approved the final manuscript.

### Conflict of Interest Statement

H-JW is founder and shareholder of Scintomics. The remaining authors declare that the research was conducted in the absence of any commercial or financial relationships that could be construed as a potential conflict of interest.
