# Supplementary material for: [68Ga]-Pentixafor PET/CT for CXCR4-Mediated Imaging of Vestibular Schwannomas
Source: Front Oncol. 2019 Jun 12;9:503. doi: 10.3389/fonc.2019.00503 (PMC6581743; doi:10.3389/fonc.2019.00503)
Supplement: Supplementary file 1 [file Table_1.DOCX]

**SUPPLEMENTARY TABLES**

**Supplemental Table 1:** Patient characteristics

| No | Tumor location | Tumor extension | Tumor development | Tumor growth | Previous therapy | Hearing impairment | | CXCR4 IRS | Ki67 (%) | Antoni type |
| --- | --- | --- | --- | --- | --- | --- | --- | --- | --- | --- |
| 1 | VS left | T3A  16x8 mm | Progressive | 2mm in 4 months | No | H3 | 9 | | 1 | A/B |
| 2 | VS right | T4B  30x35 mm | Newly diagnosed |  | No | H2 | 2 - 6 | | 1-2 | A |
| 3 | VS left | T4A  23x23 mm | Progressive |  | No | H4 |  | |  |  |
|  | VS right | T3B  16x12 mm |  | stable for 1year | Radiosurgery (2016) | H6 |  | | None |  |
| 4 | VS left | T4  26x32 mm | Progressive | 3mm in 6 months | Surgery (2008) | H6 | 6 | | 10-15 | A/B |
|  | VS right | T3A |  |  | Radiosurgery (2011) | H4 |  | |  |  |

IRS= immunoreactive score; S= schwannoma; VS = vestibular schwannoma

**Supplemental Table 2**: Individual imaging results

| No | Disease location | SUV_mean_ | SUV_max_ | Background | Bloodpool | TBR_mean_ | TBR_max_ | TBlR_mean_ | TBlR_max_ | IRS |
| --- | --- | --- | --- | --- | --- | --- | --- | --- | --- | --- |
| 1 | VS left | 3.29 | 4.05 | 0.94 | 2.56 | 2,91 | 3,59 | 1.07 | 1.32 | 9 |
| 2 | VS right | 3.23 | 4.13 | 0.99 | 2.59 | 3.26 | 4.17 | 1.25 | 1.59 | 6 |
| 3 | VS left | 3.03 | 3.78 | 0.55 | 2.21 | 5.51 | 6.87 | 1.37 | 1.71 | N/A |
|  | VS right | 3.29 | 4.05 |  |  | 5.98 | 7.36 | 1.49 | 1.83 | N/A |
| 4 | VS left | 2.56 | 3.26 | 0.90 | 2.01 | 2.84 | 3.62 | 1.27 | 1.62 | 6 |
|  | VS right | 3.17 | 3.99 |  |  | 3.52 | 4.43 | 1.58 | 1.99 | N/A |

IRS= immunoreactive score; S = schwannoma; SUV= standardized uptake value; TBlR= tumor to blood pool ratio; TBR= tumor to background ratio; VS = vestibular schwannoma
